# Enhanced EEG Emotion Recognition Using MIMO-Based Denoising and Band-Wise Attention Graph Neural Network

**DOI:** 10.3390/s26041133

**Published:** 2026-02-10

**Authors:** Yujin Ji, Do-Hyung Kim, Jungpyo Hong

**Affiliations:** 1 Information and Communication Engineering, Changwon National University, 20 Changwondaehak-Ro, Changwon 51140, Republic of Korea; 20193036@gs.cwnu.ac.kr; 2 Department of Neurology, Samsung Changwon Hospital, Sungkyunkwan University School of Medicine, Changwon 51353, Republic of Korea

**Keywords:** EEG emotion recognition, graph neural network, MIMO noise estimation and reduction, BFE-Net, subject independent

## Abstract

Electroencephalogram (EEG) signals serve as a primary input for brain–computer interface (BCI) systems, and extensive research has been conducted on EEG-based emotion recognition. However, because EEG signals are inherently contaminated with various types of noise, the performance of emotion recognition is often degraded. Furthermore, the use of a Band Feature Extraction Neural Network (BFE-Net), a state-of-the-art (SOTA) method in this field, has limitations with respect to independent band-wise feature extraction and a simplistic band aggregation process to obtain final classification results. To address these problems, this study proposes the noise-robust band-attention BFE-Net framework, aiming to improve the conventional BFE-Net from two perspectives. First, we implement multiple-input, multiple-output (MIMO)-based preprocessing. Specifically, we utilize multichannel minima-controlled recursive averaging for precise non-stationary noise covariance estimation and generalized eigenvalue decomposition for subspace filtering to enhance the signal-to-noise ratio. Second, we propose an attention-based band aggregation mechanism. By integrating a band-wise self-attention mechanism, the model learns dynamic inter-band dependencies for more sophisticated feature fusion for classification. Experimental results on the SEED and SEED-IV datasets under a subject-independent protocol show that our model outperforms the SOTA BFE-Net by 3.27% and 3.34%, respectively. This confirms that rigorous MIMO noise reduction, combined with frequency-centric attention, significantly enhances the reliability and generalization of BCI systems.

## 1. Introduction

Emotion plays a significant role in human cognitive processes, decision-making, and social interactions. Emotion recognition technology, which enables computers to accurately perceive and adaptively respond to human emotional states, has become an active research area for the advancement of brain–computer interface (BCI) and smart healthcare systems. Early studies primarily relied on externally observable non-verbal cues (e.g., facial expressions, voice, and gestures); however, these signals have inherent limitations, as they can be intentionally manipulated or suppressed [1]. In contrast, an electroencephalogram (EEG) directly reflects brain activity and is difficult to control voluntarily, making it the most reliable physiological signal for objectively understanding a user’s internal and genuine emotional state [2].

Whereas early EEG research focused on hand-crafted features, including differential entropy (DE) and power spectral density (PSD) [3], deep learning models have played a pivotal role in learning non-linear EEG patterns since the 2020s [4]. In particular, the development of graph neural networks (GNNs), which are capable of modeling functional connectivity between brain regions, has been remarkable [5]. Specifically, the Dynamical Graph Convolutional Neural Network (DGCNN) model proposed by Song et al. [6] laid the foundation for the learning of dynamic relationships between channels using adjacency matrices, while the Regularized GNN (RGNN) framework proposed by Zhong et al. [7] improved generalization performance by introducing biological and topological constraints. Furthermore, Li et al. [8] proposed the Self-Organized GNN (SOGNN) to overcome individual differences among subjects. This model autonomously learns unique brain connectivity patterns for each subject to generate dynamic graph structures, thereby surpassing the limitations of conventional methods using fixed adjacency matrices.

Recently, there have been active attempts to incorporate state-of-the-art (SOTA) techniques, including Transformers or self-supervised learning, into these GNN structures. Specifically, the Dual-Attention-Mechanism Graph Convolutional Neural Network (DAMGCN) proposed by Chen et al. [9] achieved a high degree of accuracy of over 99% in subject-dependent experiments by applying a dual attention mechanism to simultaneously learn spatial dependencies between brain regions and the importance between frequency bands. Additionally, Bi-ViTNet, proposed by Lu et al. [10], adapted the Vision Transformer (ViT) for EEG, proposing a structure that extracts and fuses spatiotemporal and frequency features in separate branches, effectively capturing long-term time-series dependencies. Furthermore, Li et al. [11] proposed a Graph-Based Multi-Task Self-Supervised (GMSS) learning framework to learn robust representations from unlabeled data, contributing to the solution of the data scarcity problem and enhancing cross-subject performance. Most recently, the Band Feature Extraction Neural Network (BFE-Net) [12] was proposed as a SOTA method that effectively extracts features from five independent frequency bands. However, it relies on a simple concatenation method when combining these features, which limits the adaptive reflection of band-wise importance, which changes rapidly according to emotional states [13]. This acts as a factor limiting the model’s expressiveness.

Despite the rapid advancement of these deep learning technologies, there are still several significant technical challenges that need to be overcome for EEG-based emotion recognition systems to be successfully deployed in real-world applications outside the laboratory. First, there are the problems of a low signal-to-noise ratio (SNR) and the non-stationarity of EEG signals. EEG signals measured at the scalp surface are very weak—in the microvolt (μV) range—and are vulnerable to various noise sources. Major noise sources include physiological artifacts (e.g., electro-oculogram, electromyogram, and electrocardiogram), environmental noise from AC power lines or surrounding electronic devices, and motion artifacts due to electrode contact instability or cable movement [14]. Various signal processing techniques, including artifact subspace reconstruction, independent component analysis (ICA), and canonical correlations analysis, as well as deep learning-based denoising approaches, have been actively applied to secure clean signals [15,16,17]. However, the effectiveness of these existing techniques is limited to specific noise types or channels, and they may fail to separate broadband artifacts that overlap with the frequency bands of brain waves. In particular, techniques like ICA have the disadvantage of being unsuitable for real-time processing due to high computational complexity. Second, there are the problems of high inter-subject variability and inefficient fusion of multi-band information. EEG signals show different patterns for the same emotional stimulus depending on the individual’s structural brain differences and cognitive state. Recent GNN-based studies, including studies investigating DGCNN [6] and RGNN [7] models, have attempted to mitigate this problem by modeling spatial relationships between electrodes, but most adopt a simple approach of merely concatenating features extracted from five major frequency bands along the channel dimension. Neuroscience studies have revealed that active frequency bands differ according to each emotional state, e.g., beta and gamma bands are activated in high-arousal states, indicating that non-linear interactions between bands contain critical information for emotion processing [18].

To fundamentally resolve these limitations, we propose Noise-Robust Band-Attention BFE-Net (NR-BA-BFE-Net), an integrated framework that simultaneously enhances the signal and strengthens feature representation. The main contributions of this study are outlined as follows:

1. Application of an MIMO noise estimation and reduction technique: We apply a multiple-input, multiple-output (MIMO) noise estimation and reduction technique to effectively remove non-stationary noise artifacts. Specifically, multichannel minima-controlled recursive averaging (M-MCRA) is applied for noise estimation, and generalized eigenvalue decomposition (GEVD)-based subspace filtering is applied for noise reduction.
2. Attention-based BFE-Net band aggregation: A single-head self-attention module is proposed to explicitly model the inter-dependency between frequency bands. Through this, the model adaptively assigns weights to frequency bands that have make considerable contributions to the determination of the current emotional state.
3. Achievement and verification of SOTA performance: In subject-independent experiments using the benchmark SEED (SJTU Emotion EEG Dataset) [19] and SEED-IV datasets, the proposed model achieved performance surpassing that of representative GNN models, including the existing DGCNN [6], RGNN [7], SOGNN [8], and BFE-Net [12] models.

## 2. Related Works

### 2.1. Theoretical Background of MIMO Signal Processing

MIMO signal processing technology has evolved in the fields of communication and speech enhancement to enhance signal quality by leveraging the spatial correlation of multichannel sensors. Early research primarily focused on single-channel techniques. Specifically, Cohen and Berdugo [20] significantly advanced spectral-domain methods by introducing the MCRA technique to handle non-stationary noise environments. In parallel, subspace approaches were developed to decompose noisy signals, with Ephraim and Van Trees [21] utilizing EVD and Hu and Loizou [22,23] extending this to GEVD for colored noise. Despite these contributions, single-channel methods face a fundamental limitation: they struggle to separate signals when the frequency bands of the target and noise overlap significantly.

To overcome this, beamforming and MIMO techniques utilizing spatial information emerged. However, traditional beamforming often requires precise direction-of-arrival estimation or complex constraints, which are difficult to maintain in diffuse or reverberant environments [24]. Consequently, interest has shifted toward linear filtering based on second-order statistics and signal presence probability (SPP) in the multichannel domain. Souden et al. [25] extended the noise estimation framework to M-MCRA, and Hong [26] further refined this by incorporating multichannel SPP into subspace filtering to enhance the SNR while reducing distortion. In this study, we adapt these proven MIMO noise reduction frameworks to the EEG signal processing domain.

### 2.2. BFE-Net

BFE-Net [12] acts as the backbone model for this study, featuring a structure that combines the powerful spatial feature learning capability of a graph convolutional network (GCN) with frequency band-specific characteristics. Specifically, this model uses DE measurementsextracted from five major frequency bands as key features [3]. DE is an indicator that represents both the complexity and energy of the signal. The extracted features undergo linear dynamical system (LDS) smoothing to reflect the temporal continuity of emotional states, converting them into smooth time-series data with suppressed sudden fluctuations.

These refined features are processed for each frequency band through an independent sub-network called BFE-Net. BFE-Net undergoes a hierarchical feature extraction process consisting largely of three stages. The first stage is the hierarchical convolutional neural network (CNN) layer. BFE-Net uses a CNN to capture temporal flows and abstract features within each EEG channel. Specifically, it is constructed by stacking blocks containing convolution layers with a kernel size of 1×5 and max pooling in three stages, with the number of filters gradually increasing from 64 to 128, then to 256. Through this, the model learns local patterns in shallow layers and high-dimensional semantic information in deep layers.

The second stage is the self-adaptive multi-graphic layer. Unlike existing GNN studies that used fixed distance-based adjacency matrices without considering inter-subject deviations, BFE-Net utilizes a mechanism to dynamically learn correlations between channels from data. For this, a modified Transformer encoder structure is used. First, multi-head attention is performed to calculate an attention map (*A*) representing the latent connection strength between channels. Simultaneously, the self-correlation matrix (*S*) of the encoded features is obtained and combined with *A* to generate an adjacency matrix (E=softmax(A+S)) containing both global and local information. In this process, a top-k sparsification strategy is applied to retain only the top *k* edges with high connection strength for each node to remove unnecessary connections and strictly improve computational efficiency.

The last stage is spatial feature aggregation via graph convolution. By combining the constructed sparse adjacency matrix (*E*) and the node features previously extracted through the CNN, spectral graph convolution operations are performed. This is defined as $H^{\prime }=\sigma \left({\hat{D}}^{-1/2}\hat{E}{\hat{D}}^{-1/2}X\Theta \right)$, where $\hat{E}=E+I$ is the adjacency matrix including self-loops, $\hat{D}$ denotes the degree matrix, Θ represents the learnable weight matrix, and σ(·) is the non-linear activation function (e.g., Leaky ReLU). Through this process, BFE-Net effectively learns spatial features reflecting the complex connectivity between brain regions.

## 3. Proposed Methods

**Figure 1:**
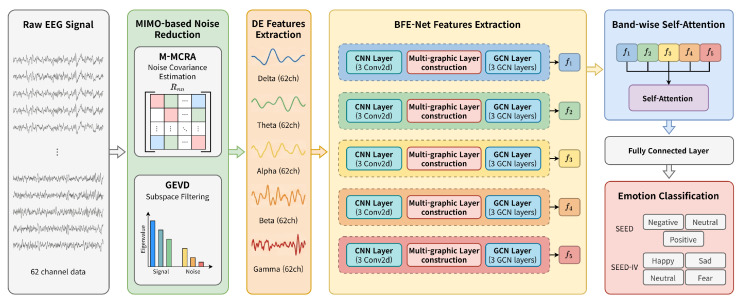
Overall framework of the proposed NR-BA-BFE-Net.

In this section, we describe, in detail, NR-BA-BFE-Net, the noise-robust subject-independent emotion recognition framework proposed in this study. The overall framework is illustrated in Figure 1. The proposed method consists of two key modules. First, a MIMO-based noise estimation and reduction module effectively removes noise from noisy EEG signals in non-stationary noise environments. Second, a band-wise self-attention-based feature fusion and classification module learns the inter-dependency between frequency bands of the reconstructed signal to enhance emotion recognition performance. The detailed operational principles of each module are described as follows.

### 3.1. MIMO Noise Estimation and Reduction

Noise inevitably introduced during the EEG signal collection process is a critical factor degrading emotion recognition performance. To address this, we apply an MIMO noise estimation and reduction technique. Specifically, we adapt the multichannel subspace filtering framework using spatio-spectral covariance matrices proposed by Hong [26] to the EEG domain. This method consists of M-MCRA for robust noise estimation and GEVD-based subspace filtering for effective noise reduction. The entire algorithm is performed in the short-time Fourier transform (STFT) domain, and the specific process is described as follows.

#### 3.1.1. M-MCRA-Based Non-Stationary Noise Covariance Estimation

The performance of noise reduction depends on the accurate estimation of the noise covariance matrix ($R_{nn}$). In this study, we use the M-MCRA algorithm proposed by Souden et al. [25], the multichannel extension of the MCRA algorithm, to perform robust estimation, even in time-varying, non-stationary noise environments. This algorithm smooths the signal power at each frequency bin (*k*) and time frame (*l*); tracks the minimum energy values of past frames; and calculates the SPP (p(k,l)), which is the probability that a signal exists in the current frame. Based on this probability, when Y(k,l) is the *M*-channel EEG input-signal vector, the M×M noise covariance matrix is recursively updated as follows:

(1) $$R_{nn}\left(k,l\right)=\alpha_{d}\left(k,l\right)R_{nn}\left(k,l-1\right)+\left[1-\alpha_{d}\left(k,l\right)\right]Y\left(k,l\right)Y^{H}\left(k,l\right),$$

where ${\left(\cdot \right)}^{H}$ denotes the Hermitian transpose operation. The smoothing coefficient ($\alpha_{d}\left(k,l\right)$) decreases when the SPP (p(k,l)) is low (in the noise section) to reflect the current observation and maintains a value close to 1 when it is high (in the signal section) to prevent the noise covariance from being updated. This adaptive update prevents sudden EEG activity (spikes) being mistaken for noise.

#### 3.1.2. Effective Subspace Decomposition Using GEVD

In general, GEVD simultaneously diagonalizes the covariance matrices of the noisy signal ($R_{yy}$) and the noise covariance ($R_{nn}$):

(2) $$R_{yy}V=R_{nn}V\Lambda ,$$

where V is the eigenvector matrix and $\Lambda =\mathrm{diag}(\lambda_{1},\ldots ,\lambda_{M})$ is the eigenvalue matrix. Since the eigenvalue ($\lambda_{i}$) represents the signal plus noise-to-noise ratio of the corresponding *i*-th eigenvector, major signal components with high SNRs are concentrated in the subspace corresponding to large eigenvalues.

#### 3.1.3. Adaptive Gain Control and Signal Reconstruction

Filtering is performed by applying an adaptive gain to each decomposed component. As in [26], the parameterized Wiener filter is applied, which adjusts the noise suppression strength (μ) according to the estimated SNR:

(3) $$g_{i}=\frac{\lambda_{i}-1}{\lambda_{i}-1+\mu }\;,$$

where $\lambda_{i}-1$ is the a priori SNR and the μ parameter is determined based on the log SNR.

- High SNR range (≥20 dB): Set μ≈1 to operate similarly to a Wiener filter, preventing large signal distortion.
- Low SNR range (<−5 dB): Set μ to 100 or more (experimentally, $\mu_{max}=1201$) to suppress the generation of musical noise and strongly remove background noise.
- Intermediate range: Smoothly vary μ through linear interpolation to ensure filtering continuity.

Finally, the filtered spectrum is calculated via $\hat{X}=V^{-H}GV^{H}Y$, where $G=\mathrm{diag}(g_{1},\ldots ,g_{N_{sig}},0,\ldots ,0)$ is the diagonal gain matrix. Here, $N_{sig}$ denotes the dimension of the signal-plus-noise subspace, and the remaining $M-N_{sig}$ zeros correspond to the noise subspace. In this process, $V^{H}$ performs signal analysis by mapping the frequency-domain data into the subspace domain; G applies the adaptive gains; and $V^{-H}$ performs signal synthesis, mapping back to the frequency domain. This operation is conducted for each frequency bin (*k*) and time frame (*l*). Once processed across all frequency bins, the time-domain signal is reconstructed via the inverse fast Fourier transform, followed by the overlap–add method. The reconstructed time-domain signal is then separated into five frequency bands according to the method described in Section 2.2, and the DE is extracted from each band. Unlike existing studies, this study does not use pre-extracted features provided by datasets but directly extracts features from signals from which noise has been removed through the MIMO technique, ensuring that the signal improvement effect of the pre-processing stage is fully reflected in the feature space. The extracted DE features undergo LDS smoothing and are used as input to the attention-based fusion module, which is the next stage.

### 3.2. Self-Attention-Based Band Aggregation

The existing BFE-Net model simply concatenates features extracted from each frequency band and inputs them into the classifier. However, this method fails to consider the non-linear inter-band dependencies and has the limitation of treating all bands as independent information.

**Figure 2:**
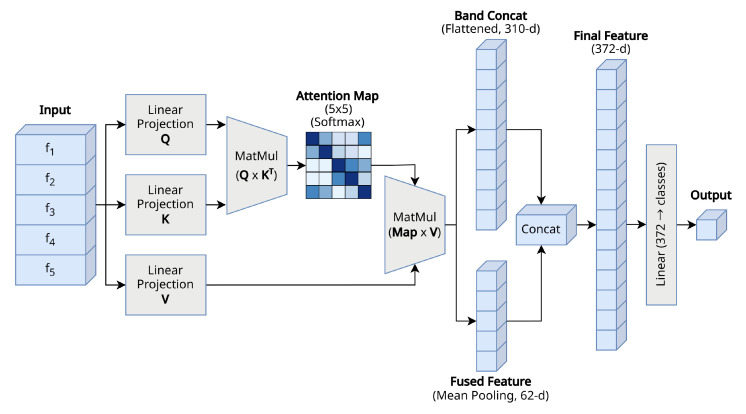
Overview of the proposed attention-based BFE-Net band aggregation process.

To address this, we propose a module that introduces a band-wise query–key–value self-attention mechanism to learn the relationships between bands and fuses the refined features reflecting this. The overall structure of the proposed band-wise attention module is shown in Figure 2. In particular, to prevent overfitting on limited EEG datasets and improve computational efficiency, we designed the model by adopting a single-head structure instead of a complex multi-head structure. This is sufficient to capture the essential interactions between the five frequency bands with only a minimal increase in parameters.

#### 3.2.1. Input Embedding and Projection

The frequency band-specific feature vectors extracted from BFE-Net are stacked to construct an input matrix ($X\in R^{N_{b}\times D}$, where $N_{b}$ denotes the number of frequency bands and *D* represents the feature dimension, corresponding to the number of channels). This input is projected into query, key, and value spaces using learnable weight matrices ($W_{Q},W_{K}\in R^{D\times d_{k}}$, and $W_{V}\in R^{D\times d_{v}}$, where $d_{k}$ and $d_{v}$ denote the dimensions of the key and value vectors, respectively). These projections are calculated as follows:

(4) $$Q=XW_{Q},\;K=XW_{K},\;V=XW_{V},$$

where Q, K, and V represent the query, key, and value matrices, which are linear transformations of the input features used to compute attention scores.

#### 3.2.2. Inter-Band Attention Map

Scaled dot-product attention is performed to measure the correlation between each frequency band. The generated attention map ($A\in R^{N_{b}\times N_{b}}$) is defined as follows:

(5) $$A=\mathrm{softmax}\left(\frac{QK^{T}}{\sqrt{d_{k}}}\right),$$

where each element ($A_{ij}$) represents the strength of the correlation between the *i*-th band and the *j*-th band and $\sqrt{d_{k}}$ is the scaling factor. Through this process, the model learns, by itself, how much to refer to information from other specific bands when processing information from a certain band.

#### 3.2.3. Feature Refinement and Fusion

The calculated attention map (A) is applied to the Value matrix (V) to obtain a new feature matrix ($X^{\prime }$) that reflects the interaction between bands. Subsequently, the dimensions are restored through linear projection:

(6) $$X^{\prime }=\mathrm{Linear}\left(AV\right).$$

Finally, to construct the comprehensive feature representation ($Z_{\mathrm{final}}$), we combine both local and global features. Specifically, the band-specific features are flattened to preserve detailed information ($F_{\mathrm{concat}}$), while a global feature vector is obtained via mean pooling to summarize the overall spectral context ($F_{\mathrm{global}}$). The final representation is formed by concatenating these two vectors:

(7) $$\begin{array}{cc} F_{\mathrm{concat}} & =\mathrm{Flatten}\left(X^{\prime }\right)\in R^{N_{b}\cdot D}, \end{array}$$

(8) $$\begin{array}{cc} F_{\mathrm{global}} & =\frac{1}{N_{b}}\sum_{i=1}^{N_{b}}X_{i}^{\prime }\in R^{D}, \end{array}$$

(9) $$\begin{array}{cc} Z_{\mathrm{final}} & =\left[F_{\mathrm{concat}};F_{\mathrm{global}}\right]\in R^{N_{b}\cdot D+D}. \end{array}$$

In this study, the $Z_{\mathrm{final}}$ generated in this way is used as the input to the final emotion classifier, designed to utilize both band-specific detailed information and organic combination information between bands.

## 4. Experiments

In this section, we present the experiments performed to verify the performance of the proposed NR-BA-BFE-Net and their results. The experiments were conducted using the SEED and SEED-IV datasets, which are representative benchmark datasets in the field of emotion recognition. We analyzed the performance in comparison with existing SOTA studies based on the subject-independent protocol. Additionally, through an ablation study, we quantitatively and qualitatively verified the impact of the proposed noise reduction module and attention mechanism on performance improvement.

### 4.1. Experimental Setup

#### 4.1.1. Datasets

To verify the performance of the model proposed in this study, we used two public datasets widely used in the emotion recognition field: SEED (SJTU Emotion EEG Dataset) and SEED-IV [19]. These datasets were constructed by Shanghai Jiao Tong University (SJTU) and are accessible via the official website (https://bcmi.sjtu.edu.cn/home/seed/, accessed on 5 January 2026).

The SEED dataset was collected from 15 subjects (7 males and 8 females; average: age 23.27 years) who watched 15 movie clips inducing three emotional states (positive, neutral, negative). Each subject participated in a total of 3 sessions at intervals of about 1 week, providing data with temporal stability. Five clips were assigned to each emotion, and the length of one clip was approximately 4 min. The experimental protocol consisted of a start hint (5 s), movie viewing (about 4 min), self-assessment (45 s), and rest (15 s). Clips were arranged so that the same emotion did not appear consecutively, considering subject fatigue. Data was collected using a 62-channel ESI NeuroScan system with the international 10–20 system placement, downsampled to 200 Hz; then, a 1–75 Hz bandpass filter was applied.

The SEED-IV dataset was built with the goal of fine-grained emotion recognition and includes four emotional states: neutral, sad, fear, and happy. Fifteen subjects with demographic backgrounds similar to those of SEED subjects participated, and 3 sessions were conducted on different dates. A total of 72 movie clips were used, designed so that 24 clips (6 per emotion) were watched in each session. The length of each video was about 2 min, which is shorter than SEED, but the classification difficulty was higher, as the ‘negative’ emotion is divided into ‘fear’ and ‘sadness’. The experimental sequence consisted of a start hint (5 s), video viewing (about 2 min), and self-assessment (45 s). Data collection and pre-processing procedures used a 62-channel system identical to that used in SEED, downsampled to 200 Hz and filtered in the 1–75 Hz band.

#### 4.1.2. Implementation Details

- STFT and Model Parameters: In the STFT process for noise reduction, a window length of 64 ms and an overlap of 50% were applied.
- Data Dimensions: The model’s input data were configured as a four-dimensional tensor of (N,62,T,5). Here, *N* denotes the total number of samples (trials), consisting of a total of 675 (15 subjects × 3 sessions × 15 trials) for the SEED dataset and 1080 (15 subjects × 3 sessions × 24 trials) for the SEED-IV dataset. The number of EEG channels is 62, and 5 represents the number of frequency bands (δ,θ,α,β,γ). For the time dimension (*T*), zero-padding was applied to unify variable trial lengths, fixing it to 265 points for the SEED dataset and 64 points for the SEED-IV dataset.
- Hyperparameters and Environment: All models in this study were implemented using the PyTorch framework (v2.5.1), and training was performed on an NVIDIA RTX A6000 GPU (NVIDIA Corporation, Santa Clara, CA, USA). The Adam optimizer was used as the optimization algorithm, with the learning rate set to 0.001, batch size to 64, and total epochs to 200. Cross-entropy loss was used as the loss function, but label smoothing of 0.1 was applied to enhance the model’s generalization performance. Additionally, the dropout rate was set to 0.1 to prevent overfitting, and leave-one-subject-out (LOSO) cross-validation was performed, that is, the process of using data from 1 out of 15 subjects as the test set and data from the remaining 14 as the training set was repeated to verify the model’s generalization performance.
- Comparative Denoising Setup: To verify the superiority of the proposed MIMO denoising, we compared it with FastICA, a widely used blind source separation algorithm. FastICA was implemented using the scikit-learn library (v1.6.1), with the number of components set to 62 (equal to the number of channels). Artifact-related independent components (ICs) were identified based on spectral characteristics (power ratio between high/low frequency bands), and the top 10% of ICs with the highest artifact scores were removed to reconstruct the clean EEG signals.

### 4.2. Experimental Results

#### 4.2.1. Comparison with State-of-the-Art Models

To verify the effectiveness of the proposed NR-BA-BFE-Net model, we compared its performance with that of widely used baseline models and SOTA emotion recognition models on the SEED and SEED-IV datasets. The comparison models include the traditional machine learning SVM method [7]; deep learning models including DAMGCN [9], DGCNN [6], RGNN [7], TANN [27], BiHDM [28], GMSS [11], and SOGNN [8]; and the current SOTA BFE-Net model [12], which is also the backbone of this study. All comparative experiments were conducted under identical data splits (LOSO) and evaluation protocols.

In the SEED dataset experiment, the proposed model achieved an average accuracy of 95.56% (standard deviation of 4.18%), demonstrating superior performance, surpassing all comparative models. Specifically, it showed a significant performance improvement compared to the traditional SVM (56.73%) and early GNN models, including DAMGCN (73.21%) and DGCNN (79.95%). It also outperformed recent advanced models, including RGNN (85.30%), TANN (84.41%), and BiHDM (85.40%). Furthermore, it exceeded SOGNN (86.81%) and the current SOTA BFE-Net model (92.29%) by 8.75% and 3.27%, respectively. Also, on the SEED-IV dataset, which has a higher difficulty, classifying four emotions, it recorded an average accuracy of 83.15% (standard deviation of 3.40%), showing the highest performance among all comparative models. It showed a large gap relative to SVM (37.79%) and consistently outperformed other deep learning models, including DAMGCN (68.22%), TANN (68.00%), BiHDM (69.03%), RGNN (73.84%), and SOGNN (75.27%). Compared to the BFE-Net backbone (79.81%), it achieved a 3.34% improvement. These results suggest that the proposed noise reduction pre-processing and band-wise attention mechanism substantially contribute to the improvement of emotion recognition performance by effectively handling noise and learning inter-band dependencies.

Table 1 focuses on comparing the accuracy of various SOTA models. However, metrics such as accuracy alone may not fully reflect the model’s reliability. Therefore, to conduct a more rigorous performance analysis, we performed a statistical verification comparison between the proposed model (NR-BA-BFE-Net) and the backbone model (BFE-Net) re-implemented under the identical experimental conditions for a fair comparison. The results are shown in Table 2. Here, we measured not only accuracy but also the Matthews Correlation Coefficient (MCC) and F1 score to evaluate the balance of classification performance. In addition, a paired *t*-test was performed to verify the statistical significance of the performance improvement.

#### 4.2.2. Ablation Study

To verify the individual contributions of the two key modules proposed in this study, the noise reduction module (NR) and the band-wise attention module (BA), we conducted an ablation study. We analyzed the performance of three variants: (1) BA-BFE-Net (w/o NR), which uses the BA module but preprocesses data using standard LDS smoothing without the proposed MIMO noise reduction; (2) BA-BFE-Net (w/FastICA), which applies standard FastICA for denoising instead of the proposed MIMO method; and (3) NR-BFE-Net (w/o BA), which applies the proposed MIMO noise reduction but uses simple concatenation for band fusion instead of the BA module. The results are summarized in Table 3.

The experimental results demonstrate that both modules contribute to performance improvement. Notably, all three variants presented in Table 3 showed different performance characteristics compared to the full proposed model (NR-BA-BFE-Net) presented in Table 1. Specifically, even without the NR module, BA-BFE-Net (w/o NR) achieved higher accuracy than the backbone model, indicating that the attention-based weighting strategy is effective. When FastICA was applied (BA-BFE-Net (w/FastICA)), performance improved to 95.08% (SEED) and 80.46% (SEED-IV), indicating that artifact removal is important for improving performance. However, the proposed MIMO-based full model (NR-BA-BFE-Net, Table 1) showed higher performance than FastICA, achieving 95.56% and 83.15%. This implies that the proposed MIMO denoising removes noise while preserving critical emotional information better than the conventional FastICA method. Similarly, NR-BFE-Net (w/o BA), which only applies the proposed MIMO noise reduction, also outperformed the backbone configuration, indicating that MIMO-based noise reduction enhances signal quality. However, the best performance was obtained when both modules were integrated, validating the synergistic effect of the proposed framework.

In addition, for the analysis of band-wise feature fusion strategies, we compared the performance of the proposed single-head self-attention-based fusion method with various fusion strategies, including scalar weight, MLP fusion, and multi-head self-attention. The results are shown in Table 4.

The single-head self-attention mechanism showed higher performance than simple learnable scalar weighting and additive attention (attention score calculated using MLP). Although additive attention can learn non-linear relationships between bands, it was confirmed that the self-attention method proposed in this study is more effective in capturing inter-band dependencies. Interestingly, multi-head attention showed lower performance than the single-head variant on both the SEED and SEED-IV datasets. This observation can be attributed to the characteristics of the feature space relative to the number of frequency bands. In our framework, the input to the attention module consists of a sequence of 5 frequency bands, each with a feature vector of 62 dimensions (matching the number of EEG channels). Multi-head attention is typically beneficial when the sequence is long and diverse subspaces need to be captured simultaneously. However, given the extremely short sequence length of 5, the single-head structure is sufficient to capture the global dependencies between bands. Dividing the 62-dimensional features into multiple heads (e.g., 4 heads) results in very low-dimensional subspaces (approx. 15 dimensions) for each head, which may fail to capture the rich semantic information contained in the original features, potentially leading to suboptimal representation learning. Therefore, the single-head structure adopted in this study is a suitable and efficient architecture for learning relationships between the limited number of EEG bands.

#### 4.2.3. Qualitative Analysis and Visualization

**Figure 3:**
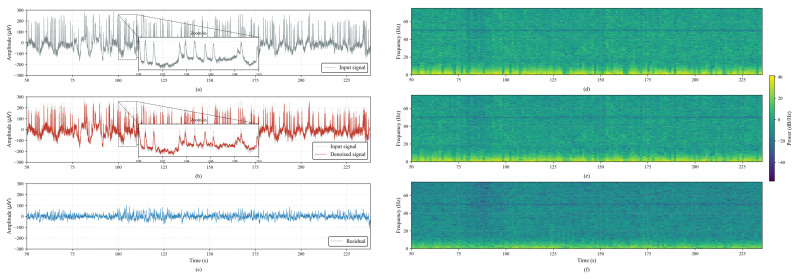
Visualization of MIMO-based noise reduction performance. (**a**) Input waveform (original signal). (**b**) Denoised waveform after applying the proposed module (red), overlaid with the original signal (gray). (**c**) Residual waveform, defined as the removed component (input–denoised). (**d**) Spectrogram of (**a**) input waveform. (**e**) Spectrogram of (**b**) denoised waveform. (**f**) Spectrogram of (**c**) residual waveform.

To verify the effect of the proposed noise reduction module, we applied the proposed MIMO-based denoising to the multichannel EEG signals (62 channels) and visualized a representative single-channel waveform for qualitative inspection. Figure 3a–c show the EEG waveforms, where Figure 3a presents the input signal and Figure 3b presents the denoised waveform after applying the proposed module. As highlighted in the zoomed-in region of Figure 3b, abrupt spike-like fluctuations observed in the input signal were attenuated, while the overall temporal structure of the EEG waveform was preserved. Furthermore, the residual signal (Figure 3c), defined as the difference between the input and denoised signals (Residual=Input−Denoised), provides an intuitive view of the components reduced by the proposed module. The residual waveform exhibits relatively low-amplitude fluctuations compared to the input signal and shows irregular patterns, suggesting that the proposed method primarily attenuates noise-related components while preserving meaningful EEG activity. To further examine the residual characteristics in the time–frequency domain, we additionally present single-channel spectrograms of the input, denoised, and residual signals in Figure 3d–f. The input spectrogram (Figure 3d) shows relatively high spectral energy distributed across a wide frequency range, whereas the denoised spectrogram (Figure 3e) exhibits a reduced spectral energy level with a more stable spectral structure. Moreover, the residual spectrogram (Figure 3f) remains at a comparatively low power level, qualitatively confirming that the proposed module effectively suppresses noise-related components in the original signal. However, due to the absence of ideal ground-truth signals in real-world datasets, direct quantitative evaluation using metrics such as the SNR was considered beyond the scope of this study. Therefore, we focused on qualitative verification through waveform comparison, residual analysis, and spectrogram inspection, and we plan to conduct precise quantitative performance evaluation as future work.

**Figure 4:**
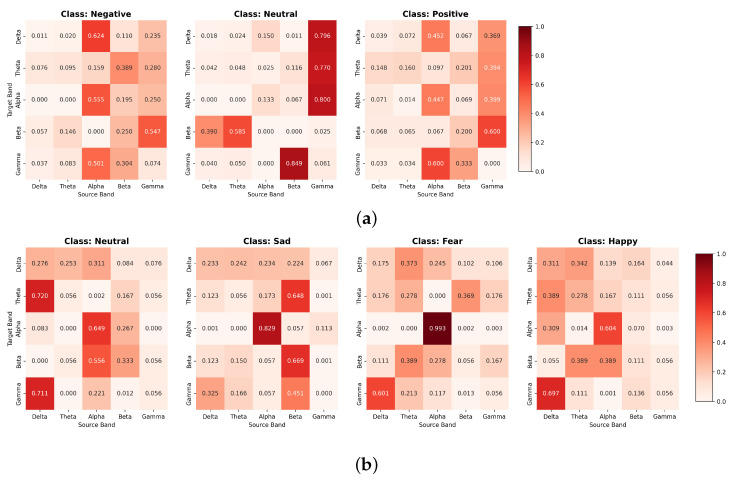
Visualization of band attention map by emotion. (**a**) SEED dataset, (**b**) SEED-IV dataset. (Heatmap values indicate the weight contributed to the target band by the source band).

Next, we analyzed how the single-head self-attention mechanism learned the interaction between each frequency band through attention-map visualization. The learned attention weights are visualized as heatmaps in Figure 4a,b, which represent the average attention maps for each emotion of a representative subject (subject 1) in the SEED and SEED-IV datasets. Here, the x-axis represents the source band (key), and the y-axis represents the target band (query). The results show that the inter-band attention patterns are clearly distinguished according to the emotional state in both datasets. In the SEED dataset (Figure 4a), the neutral state shows strong attention toward the gamma band as a source band from multiple target bands (e.g., alpha→gamma and delta→gamma). In addition, positive and negative emotions exhibit noticeable cross-band interactions involving the alpha and gamma bands, indicating that emotion-dependent frequency-band relationships are learned by the proposed attention module. In the SEED-IV dataset (Figure 4b), strong self-attention in the alpha band (0.99) is observed in the fear state, suggesting that the alpha-band representation is highly emphasized under this condition. In the sad state, attention is strongly concentrated toward the beta band, showing prominent cross-band interactions such as theta-to-beta attention. These results visually demonstrate that the proposed band-wise attention module effectively captures emotion-specific frequency-band interaction patterns.

**Figure 5:**
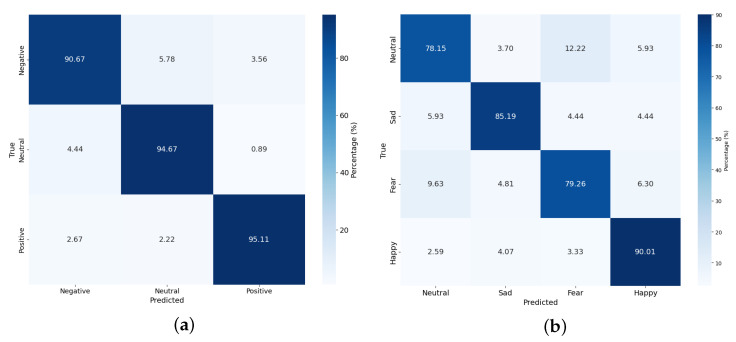
Confusion Matrix for (**a**) SEED and (**b**) SEED-IV datasets.

Finally, confusion matrix analysis was performed. Figure 5 shows the model’s confusion matrix for the SEED and SEED-IV datasets. The values of the diagonal elements are significantly higher than other off-diagonal elements (SEED: >95%, SEED-IV: >83%), indicating that the proposed model shows balanced classification performance for all emotion classes. In particular, although there was some notable misclassification between ’neutral’ and ’fear’ in the SEED-IV dataset, this suggests that distinguishing between neutral states and certain emotional arousal states remains a challenging task. Overall, it was confirmed that the proposed model effectively mitigates this ambiguity through the attention mechanism.

### 4.3. Computational Complexity Analysis

While ensuring high signal quality is paramount, evaluating computational efficiency is equally vital for practical BCI applications. ICA-based denoising (FastICA [29] and Extended Infomax [30]) typically relies on iterative optimization algorithms to estimate the unmixing matrix, where the per iteration cost is approximately proportional to $M^{2}N$ and $M^{3}$, leading to an overall complexity of $O\left(I\left(M^{2}N+M^{3}\right)\right)\approx O\left(IM^{2}N\right)$, where *M* denotes the number of EEG channels, *N* denotes the number of temporal samples per trial, and *I* denotes the number of ICA iterations required for convergence. On the other hand, the proposed MIMO-based preprocessing utilizes algebraic approaches including STFT and GEVD-based subspace decomposition, and its complexity is dominated by the GEVD process ($O(TKM^{3})$) and STFT/ISTFT (O(NMlogL)), expressed as $O\left(TKM^{3}+NM\log L\right)\approx O\left(NM^{3}\right)$, where *L* denotes the FFT length used in the STFT, *T* denotes the number of time frames, and *K* denotes the number of frequency bins (for real-valued FFT, K=L/2+1). Moreover, under 50% overlap ((H=L/2)), T≈N/H and K≈L/2, yielding TK≈N and, thus, $O\left(TKM^{3}\right)\approx O\left(NM^{3}\right)$. To compare the practical computational burden, the iteration count (*I*) of FastICA was empirically measured using scikit-learn on the entire SEED dataset, yielding an average of I=98.22. For Extended Infomax, the iteration count was measured using the EEGLAB toolbox [31] and averaged over all trials of a single subject, resulting in I=346.5 (Table 5). The analysis shows that the iteration count of FastICA is comparable to the number of EEG channels used in this study (M=62). Consequently, the computational complexity of the proposed MIMO method ($O(NM^{3})$) is at a level similar to that of standard ICA algorithms ($O(IM^{2}N)$), demonstrating that the proposed method maintains a computational burden comparable to that of widely used ICA algorithms, thereby ensuring its practical applicability.

## 5. Conclusions

In this study, to increase the accuracy and reliability of EEG-based emotion recognition, we proposed an integrated framework combining MIMO noise estimation and reduction techniques and a band-specific attention mechanism. This technique, applied in the pre-processing stage, consists of precise MIMO noise estimation via M-MCRA and noise reduction via GEVD-based subspace filtering, effectively removing non-stationary noise and artifacts to improve signal quality. In addition, the single-head self-attention-based feature fusion module elaborately captured the physiological characteristics of emotions by deeply learning the interactions between five frequency bands.

As a result of the experiments, the proposed model achieved excellent accuracy on the SEED and SEED-IV datasets, with performance surpassing that of existing SOTA models. In particular, through confusion matrix analysis, it was verified that balanced classification performance was shown across all emotion classes. These results suggest that the methodology of this study can achieve high generalization performance and reliability, even in a subject-independent real-world BCI environment.

However, in this study, due to the absence of clean ground-truth signals due to the nature of real datasets, there were limitations in quantitative evaluation (SNR, etc.) of noise reduction performance. In future research, we plan to quantitatively prove the signal improvement effect of the pre-processing module by constructing sophisticated simulation data and to further advance recognition performance by fusing multi-modal data (e.g., eye movements), in addition to EEG. Also, the DE feature currently used effectively reflects the non-linearity of EEG, but sensitivity to temporal changes may be somewhat lacking. Therefore, in the future, we plan to additionally apply and compare various feature extraction techniques that can reflect the dynamic characteristics of signals, including dynamic differential entropy (DDE).

Consequently, the NR-BA-BFE-Net model proposed in this study is research that has raised the practicality of EEG-based emotion recognition systems to the next level by combining pre-processing technology that guarantees stable performance, even in noisy environments, and attention modeling that precisely reflects the physiological characteristics of brain waves. This approach is expected to be utilized as a core fundamental technology for accurately and robustly identifying user states in various healthcare and metaverse interface fields based on EEG, as well as emotion recognition, in the future.

## Figures and Tables

**Table 1:** Comparison of average accuracy and standard deviation by model for SEED and SEED-IV datasets. The best-performing results are highlighted in bold.

Model	SEED	SEED-IV
SVM [7]	56.73% ± 16.29%	37.79% ± 12.52%
DAMGCN [9]	73.21% ± 8.35%	68.22% ± 7.03%
DGCNN [6]	79.95% ± 9.02%	-
RGNN [7]	85.30% ± 6.72%	73.84% ± 8.02%
TANN [27]	84.41% ± 8.75%	68.00% ± 8.35%
BiHDM [28]	85.40% ± 7.53%	69.03% ± 8.66%
GMSS [11]	86.52% ± 6.22%	73.48% ± 7.41%
SOGNN [8]	86.81% ± 5.79%	75.27% ± 8.19%
BFE-Net [12]	92.29% ± 4.65%	79.81% ± 4.11%
**NR-BA-BFE-Net (Proposed)**	**95.56% ± 4.18%**	**83.15% ± 3.40%**

**Table 2:** Detailed performance comparison and statistical analysis between the backbone and proposed model. (*p*-value < 0.05 indicates statistical significance).

Dataset	Model	Accuracy	MCC	F1 Score	*p*-Value
SEED	BFE-Net	92.59% ± 5.05%	89.40% ± 7.08%	92.37% ± 5.39%	-
	NR-BA-BFE-Net	95.56% ± 4.18%	93.55% ± 5.96%	95.41% ± 4.28%	0.032
SEED-IV	BFE-Net	80.65% ± 2.46%	74.65% ± 3.26%	80.40% ± 2.58%	-
	NR-BA-BFE-Net	83.15% ± 3.40%	77.90% ± 4.49%	82.99% ± 3.55%	0.014

**Table 3:** Ablation study on key modules (NR: noise reduction; BA: band attention).

Dataset	Model	Accuracy	MCC	F1 Score
SEED	BA-BFE-Net (w/o NR)	93.48% ± 3.19%	90.71% ± 4.45%	93.33% ± 3.39%
	BA-BFE-Net (w/FastICA)	95.08% ± 4.30%	92.85% ± 6.19%	94.99% ± 4.31%
	NR-BFE-Net (w/o BA)	93.17% ± 5.13%	90.27% ± 7.02%	92.70% ± 5.76%
SEED-IV	BA-BFE-Net (w/o NR)	81.85% ± 4.74%	76.13% ± 6.22%	81.63% ± 4.83%
	BA-BFE-Net (w/FastICA)	80.46% ± 3.23%	74.21% ± 4.32%	80.30% ± 3.19%
	NR-BFE-Net (w/o BA)	82.13% ± 3.24%	76.57% ± 4.30%	81.95% ± 3.30%

**Table 4:** Ablation study on fusion strategies.

Fusion Method	SEED	SEED-IV
Learnable Scalar Weighting	93.65% ± 4.81%	81.30% ± 2.95%
Additive Attention	94.44% ± 3.95%	80.46% ± 3.30%
Single-Head Self-Attention	95.56% ± 4.18%	83.15% ± 3.40%
Multi-Head Self-Attention	93.02% ± 4.56%	81.94% ± 4.30%

**Table 5:** Complexity comparison (Big-O and iterations).

Method	Complexity (Big-O)	Iterations (*I*)
Extended Infomax	$O\left(I\left(M^{2}N+M^{3}\right)\right)\approx O\left(IM^{2}N\right)$	346.5
FastICA	$O\left(I\left(M^{2}N+M^{3}\right)\right)\approx O\left(IM^{2}N\right)$	98.22
MIMO	$O\left(TKM^{3}+NM\log L\right)\approx O\left(NM^{3}\right)$	-

## Data Availability

Publicly available datasets were analyzed in this study. This data can be found here: SEED dataset at https://bcmi.sjtu.edu.cn/home/seed/seed.html (accessed on 5 January 2026) and SEED-IV dataset at https://bcmi.sjtu.edu.cn/home/seed/seed-iv.html (accessed on 5 January 2026).
